# A Random Forests Framework for Modeling Haplotypes as Mosaics of Reference Haplotypes

**DOI:** 10.3389/fgene.2019.00562

**Published:** 2019-06-27

**Authors:** Pierre Faux, Pierre Geurts, Tom Druet

**Affiliations:** ^1^Unit of Animal Genomics, GIGA-R, Faculty of Veterinary Medicine, University of Liège, Liège, Belgium; ^2^Department of Electrical Engineering and Computer Science, Montefiore Institute, University of Liège, Liège, Belgium

**Keywords:** random forests, supervised classification, haplotype mosaic, imputation, extra-trees

## Abstract

Many genomic data analyses such as phasing, genotype imputation, or local ancestry inference share a common core task: matching pairs of haplotypes at any position along the chromosome, thereby inferring a target haplotype as a succession of pieces from reference haplotypes, commonly called a mosaic of reference haplotypes. For that purpose, these analyses combine information provided by linkage disequilibrium, linkage and/or genealogy through a set of heuristic rules or, most often, by a hidden Markov model. Here, we develop an extremely randomized trees framework to address the issue of local haplotype matching. In our approach, a supervised classifier using extra-trees (a particular type of random forests) learns how to identify the best local matches between haplotypes using a collection of observed examples. For each example, various features related to the different sources of information are observed, such as the length of a segment shared between haplotypes, or estimates of relationships between individuals, gametes, and haplotypes. The random forests framework was fed with 30 relevant features for local haplotype matching. Repeated cross-validations allowed ranking these features in regard to their importance for local haplotype matching. The distance to the edge of a segment shared by both haplotypes being matched was found to be the most important feature. Similarity comparisons between predicted and true whole-genome sequence haplotypes showed that the random forests framework was more efficient than a hidden Markov model in reconstructing a target haplotype as a mosaic of reference haplotypes. To further evaluate its efficiency, the random forests framework was applied to imputation of whole-genome sequence from 50k genotypes and it yielded average reliabilities similar or slightly better than IMPUTE2. Through this exploratory study, we lay the foundations of a new framework to automatically learn local haplotype matching and we show that extra-trees are a promising approach for such purposes. The use of this new technique also reveals some useful lessons on the relevant features for the purpose of haplotype matching. We also discuss potential improvements for routine implementation.

## Introduction

**FIGURE 1:**
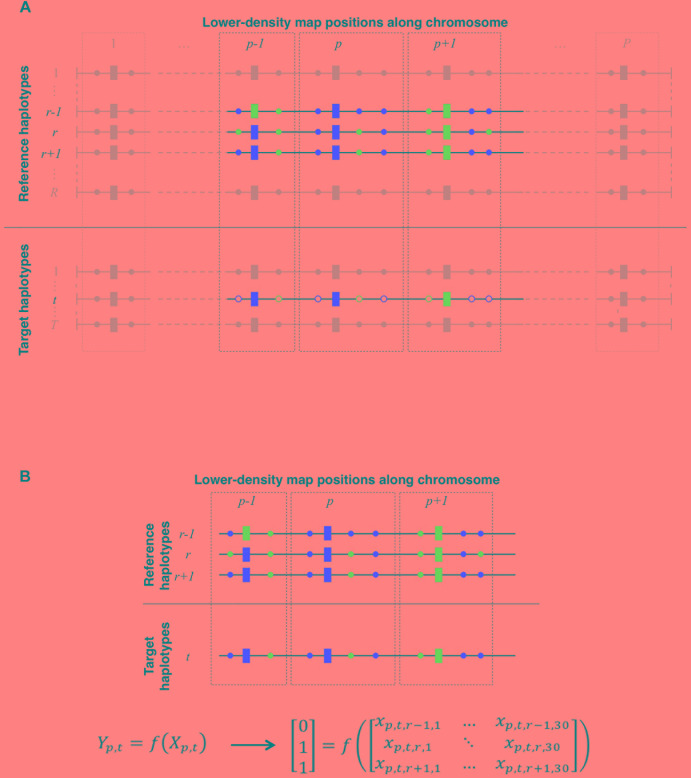
**(A)** Schematic representation of local haplotype matching. Each horizontal line features a whole-chromosome haplotype (phased from red/blue bi-allelic genotypes), to be locally matched (target) to other haplotypes (reference). Both target and reference haplotypes have *P* positions observed on the LD map (rectangles) whereas reference haplotypes may be also observed on a HD map (circles, plain color if observed), thereby allowing imputation of the target haplotype. For a given target haplotype *t*, the question is to find which one of the *R* reference haplotypes matches the best with *t*, in the neighborhood of LD position *p* (delimited by dotted lines). Here, at positions *p* – 1, *p,* and *p* + 1, *t* perfectly matches with *r* and *r* + 1, however, *t* perfectly matches on HD positions only with *r* + 1. Therefore, locally matching haplotypes in such case comes down to match *t* to *r* + 1 rather than to *r*. **(B)** Translating local haplotype matching into machine-readable language. At a LD map position *p*, a target haplotype *t* can be matched to *R* reference haplotypes. Because target haplotypes are also observed on the HD map, we measure the success of each of the *R* local matches by computing the similarity between *t* and each reference haplotypes on HD markers that are closer to the LD position *p* than to any other LD position. Reference haplotypes returning the highest similarity with *t* earn a 1 (*success*) in the observation vector *Y*_p,t_ whereas others earn a 0 (*fail*). Additionally, we compute a vector *X*_p,t,r_. of observed features (see Table 2) for any reference haplotype *r*. The machine learns how to discriminate *successes* from *fails* in *Y*_p,t_ according to features in *X*_p,t_. Here, on HD markers closest to *p*, the target haplotype *t* is identical to reference haplotypes *r* and *r* + 1. This is therefore the maximum similarity observable for haplotype *t* at position *p*. Thus, both reference haplotypes *r* and *r* + 1 earn a *success* (*Y*_p,t,r_ = *Y*_p,t,r + 1_ = 1) whereas any other reference haplotype less similar to *t* (e.g., *r* – 1) earns a *fail* (*Y*_p,t,r − 1_ = 0).

Modeling a target haplotype as a succession of segments from other haplotypes (referred to as *reference* or *template* haplotypes) is a common issue and a primary step in various genotype data analyses such as genotype imputation (e.g., in Burdick et al., 2006; Li et al., 2006; Marchini et al., 2007; Howie et al., 2009; Daetwyler et al., 2011; Sargolzaei et al., 2014) often coupled with phase reconstruction, local ancestry inference (e.g., in Price et al., 2009; Baran et al., 2012; Maples et al., 2013), estimation of identity-by-descent between segments (Druet and Farnir, 2011), or even clustering (e.g., in Su et al., 2009; Lawson et al., 2012). To describe this modeling procedure, it is commonly written that target haplotypes are modeled as a mosaic of reference haplotypes (e.g., Burdick et al., 2006; Baran et al., 2012). At any map position along the chromosome, the issue is to find which reference haplotype matches the target haplotype best (Figure 1A). Answering this question, for instance in the particular case of genotype imputation, allows to infer the target haplotype on a higher density map, on which the reference haplotypes were observed. Several sources of information are useful to address this question. Many methods (Li et al., 2006; Scheet and Stephens, 2006; Howie et al., 2009; Price et al., 2009) only take into consideration the linkage disequilibrium information. Family information can also be a trustful source, when available at large scale, for instance in livestock (Daetwyler et al., 2011; Sargolzaei et al., 2014). Linkage information (Burdick et al., 2006; Druet and Farnir, 2011; Sargolzaei et al., 2014) is a third potential source of information to locally match haplotypes. Common methods to address this question are usually either based on hidden Markov models (HMM-based methods; see Scheet and Stephens, 2006 for a general model) or rely on a set of deterministic rules (heuristic methods, e.g., based on long-range segments shared between individuals as in Kong et al., 2008).

The development of the latter type of methods, heuristics, could be described as the iterative repetition of two main steps. First, during a conception step, the human operator identifies relevant variables and uses them in a set of rules. Then, during a validation step, the proposed heuristic is tested. If the validation does not return the desired efficiency, then the human operator adjusts the heuristic in the conception step and validates it again. Conception and validation steps would therefore be repeated back and forth until enough efficiency is reached. Defining in these terms the development of a heuristic method for the issue of local haplotype matching makes it an attractive problem for a class of machine learning methods known as *supervised classification*. In such a learning framework, the classifier is fed with data containing both explicative variables (hereafter referred to as *features*, as this denomination prevails in the machine learning community) and their classification (variable to explain, also referred to as *labels*). Then, the data is repeatedly partitioned between a learning sample, on which the classifier performs the conception step, and an independent testing sample, on which the classifier assesses the efficiency of the method. We recommend to readers the review by Libbrecht and Noble (2015) for a detailed glossary as well as clear explanations about the terms used in machine learning.

Additionally, supervised classification also allows combining automatically different sources of information with flexibility. Such aspects make it interesting for locally matching haplotypes: although most of the HMM-based methods (using models similar to Scheet and Stephens, 2006) only rely on haplotype similarity, other methods (e.g., Druet and Georges, 2010) can reach higher efficiency by integrating linkage information. Also, supervised classification returns the importance of any explicative variable as a useful by-product for improving other methods. Because of these advantages, Maples et al. (2013) have already used supervised classification to address a specific problem of local haplotype matching – local ancestry inference. In their approach (RFMix), these authors implemented a random forests (RF) classifier which uses positions along the genetic map as the features.

Here, our main objective is to describe a new learning framework to locally match haplotypes using an extremely randomized trees classifier (*extra-trees*, a particular type of RF method; see Geurts et al., 2006). In this framework, a supervised classifier learns from a large collection of examples what are the relevant features to take into consideration when searching for the reference haplotype that best locally matches a target haplotype and how to combine them. We show that the learning framework accurately finds the best local matches by comparing it to a state-of-the-art HMM-based framework equivalent to IMPUTE2 (Howie et al., 2009). We eventually discuss the main findings of our framework in terms of the importance of features and propose improvements.

## Materials and Methods

### Long-Range Haplotype Pre-phasing

All computations and results presented here come from genotypes (for the lower-density map) and WGS (for the higher density map) of the first bovine autosome (BTA1) of 91 dairy cattle from New Zealand (67 bulls and 24 cows; partitioned as 36 Holstein-Friesian, 24 Jersey and 31 crossbred individuals). All individuals have been genotyped with the BovineSNP50k (v1 and v2) genotyping array from Illumina. A total of 2,321 SNPs remained for BTA1 after cleaning the initial data as described in Faux and Druet (2017) and shaped a lower density map, later referred to as the “LD map.” Those genotypes were phased using both linkage disequilibrium and family information.

Besides genotyping, all individuals were sequenced at high coverage (15× or more). Details about sequencing and downstream filters can be found in the study by Charlier et al. (2016). A map of 328,045 SNPs from chromosome BTA1 was obtained using stringent filtering rules (described in Faux and Druet, 2017); this map is later referred to as the *higher-density* (HD) map and includes the 2,321 SNPs from LD map. Using stringent rules allowed reducing the proportion of noise in our data set (e.g., assembly errors, false variants, incorrect genotypes, or phasing errors). These stringent filtering rules include, among others: (1) comparisons to other sets of WGS SNPs (markers are kept if they were observed in other available bovine WGS datasets and if they displayed correct Mendelian segregation in another WGS dataset), (2) removal of genomic regions because of a high suspicion of incorrect mapping, and (3) removal of SNPs based on additional rules for error detection.

The HD map was then phased by the two-step method outlined in Faux and Druet (2017). In a few words, this method exploits the haplotypes estimated on a genotyped population much larger (∼58,000 dairy cattle individuals from New Zealand – more details in Faux and Druet, 2017) than the 91 sequenced individuals used in the present study. Therefore, the resulting 182 haplotypes are very accurate: 99.72% of the SNPs whose phasing can be assessed using Mendelian segregation rules were proved to be assigned to their correct parental origin. Based on these results, we consider these haplotypes as the *true* haplotypes in the present study.

### Criteria for Methods Comparison

In this study, we detail a framework for automatic learning of rules to locally match haplotypes and we compare it to an HMM-based method designed for the same purpose. That comparison method is inspired from Howie et al. (2009) and fully described in the section “Hidden Markov Model for Local Haplotype Matching.” In order to quantify the ability of each method to accurately achieve this purpose, we partition the full set of 182 haplotypes in reference and target panels. Haplotypes in the target panel are observed only on the LD map whereas those in the reference panel are observed on both LD and HD maps. Any given target haplotype is locally matched to all reference haplotypes on the LD map. Then based on the quality of these local matches, the target haplotype is inferred as a mosaic of the reference haplotypes (which are observed on the HD map).

The first and main criterion to compare methods is, for any target haplotype, the difference between the inferred and the true haplotypes on the HD map, measured by the metric *e*_A_ as the proportion of the 328,045 SNPs whose inferred allele is different from the true allele. Such haplotype-based comparison is possible because we consider the phased haplotypes as correct enough to be the true ones. To get rid of the remaining phasing errors in method comparisons, we used a second criterion based on genotypes rather than on haplotypes: imputation reliability (*r*^2^), measured, for any SNP specific to the HD map, as the squared correlation between imputed and observed genotypes of all target individuals (see section “Cross-Validation Plan,” for partitioning the population in reference and target). Details are given in the next sections on how imputation is performed within the random forests framework and the HMM. We also observed the number of switches from a reference haplotype to another one. Such an observation does not reflect the ability of the methods to reach their objective but provides information on their properties (how many segments from reference haplotypes does the method use when modeling a target haplotype as a mosaic).

### Cross-Validation Plan

**FIGURE 2:**
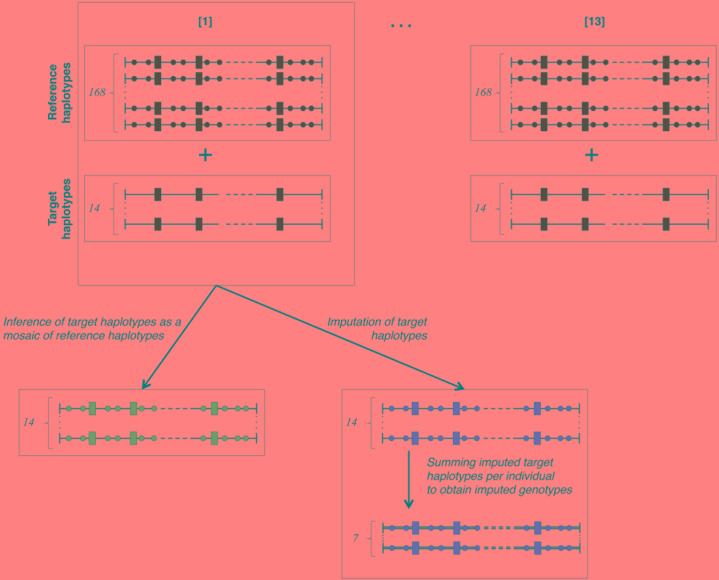
Cross-validation plan. The total number (182) of haplotypes in our study is divided into 13 cross-validation groups containing 168 reference haplotypes known on both LD (rectangles) and HD (circles) maps and 14 target haplotypes only known on the LD (rectangles) map. The 14 target haplotypes are each inferred as a mosaic of segments from reference haplotypes and simultaneously imputed from reference haplotypes. Summing per individual the imputed haplotypes returns seven imputed genotypes for each cross-validation group. Green-faced shapes are known (true haplotypes), blue-faced are modeled as mosaic of references and red-faced are imputed.

The cross-validation plan is outlined in Figure 2. In order to obtain numerous cross-validation groups (of uniform size) while keeping a training set of a reasonable size, we have chosen to partition the 91 individuals in thirteen groups of cross-validation (13-fold cross-validation scheme – as detailed in section 7.10.1 of Hastie et al., 2017). In each one of them, fourteen target haplotypes (i.e., those of seven target individuals) are inferred as mosaics of 168 reference haplotypes (i.e., those of 84 reference individuals). Then, the missing genotypes of the seven target individuals are imputed on the HD map. The seven animals forming each batch are randomly picked among the 91 animals. In each of these cross-validation groups, the fourteen target haplotypes are simultaneously imputed and modeled as a mosaic of segments from reference haplotypes. The fourteen imputed haplotypes are then summed pairwise (per individual) to obtain seven imputed genotypes per HD marker. Once cross-validation is achieved over all the 13 groups, there are 182 target haplotypes inferred as mosaic of reference haplotypes and 91 imputed genotypes per HD marker. Comparison criteria *e*_A_ and *r*^2^ are then measured respectively on all the inferred target haplotypes and on all HD markers for 91 imputed genotypes.

### Machine Learning Framework for Local Haplotype Matching

#### General Framework

The purpose of local haplotype matching is to answer the following question (see Figure 1A): at a given position *p* along the chromosome, which of the *R* reference haplotypes would match at best with a given target haplotype *t*? Answering that question for the *P* map positions leads to the reconstruction of haplotype *t* as a mosaic of segments picked from the *R* reference haplotypes. Hereafter, we detail a framework that makes this question answerable using an automatic classifier.

Let us consider a target haplotype *t* and a panel of *R* reference haplotypes. Both are observed on two maps of different densities (LD and HD maps). At a given position *p*, we assume that *t* could be matched to *R* haplotypes (see Figure 1B); therefore, among the *R* possible local matches with *t*, we expect at least one to be better than others. To find this one out, we first compute a local difference, denoted *d*_p,t,r_, for any couple of haplotypes *t* (target) and *r* (reference) at position *p*. Considering all the HD positions for which *p* is the closest position on the LD map, the difference between *r* and *t* is computed as the number of these HD positions that carry a different allele between *r* and *t*. This difference is basically a measure of local similarity between haplotypes. Once all the *R* differences are obtained, a *success* score (1) award the reference haplotype(s) showing the lowest difference with *t* whereas other reference haplotypes earn a *fail* score (0), returning thus a *r*-long scoring vector **y**_p,t_ whose elements are computed as follows:

$$y_{p,t,r}=\{\begin{array}{c} 1,\text{ if}\frac{\left(d_{p,t,r}-\min(d_{\text{p},\text{t}})\right)}{n_{\text{HD}}}\leq 0.01\\ 0,\text{ otherwise} \end{array}$$

where *n*_HD_ is the number of HD positions for which *p* is the closest LD position. As expressed in the previous formula, more than one reference haplotype may earn a *success* score: obviously all those whose local difference with *t* is the lowest, but also those whose local difference with *t* is very close to the lowest local difference (arbitrarily defined as less than 1% of difference in similarity with the best matching haplotype).

The machine learning task is to build up a classifier that discriminates the best reference haplotype from others. For this purpose, we have to feed the classifier with observations on the same features for all the *R* reference haplotypes. There are many featured observations that may prove to be helpful, e.g., the genetic relationship between haplotype *t* and any reference haplotypes or the fact that a long identical segment is shared by *t* and a given reference haplotype on the LD map. Those features can be specific to one map position (as the latter example) or not (as the former one). Measuring these features for all the *R* reference haplotypes at all the *P* LD positions shapes a *R*-by-*P*-by-*N* collection of observations (where *N* is the number of features). Each observation of the learning sample from which to train the classifier is therefore a vector **x**_p,t,r_ of observed features that corresponds to a specific triplet (*p*,*t*,*r*) with *p* a LD position, *t* a target haplotype, and *r* a reference haplotype. The number of observed features defines the length of each vector **x**_p,t,r_. Following the terminology of the machine learning community the *success*|*fail* score that corresponds to each observation is hereafter referred to as the *label*. The learning sample thus contains labeled observations, whereas samples with data to predict would contain unlabeled observations (i.e., observed features for each point *p,t,r* but not their score, which remains to predict). The goal of the machine learning algorithm is now to exploit observations in the learning sample and their labels in order to build up a classifier that efficiently discriminates *successes* from *fails*.

#### Specific Implementation With Extra-Trees Classifier

The following section details the implementation of the general framework specifically achieved to address the second research objective of this study, namely, to compare the efficiency of the machine learning classifier to locally match haplotypes to an HMM-based method.

Supervised classification is here achieved using the extremely randomized trees method (*extra-trees* hereafter), an ensemble method based on random forests (originally proposed by Geurts et al., 2006). Growing a decision tree works by gathering labeled observations showing identical values of features into a node and then splitting the node if a substantial proportion of these observations have distinct labels (*success* or *fail* in our specific case). The growing process can be illustrated with the theoretical example in Table 1: the observations listed in that table are considered as pertaining to the same node of a decision tree. In that theoretical example, we consider two features: the length of a segment shared by target and reference haplotypes (LSS) and the genomic relationship between target and reference gamete on the current chromosome (GENGc). A node split gathering all observations that have a value of LSS greater than 1,000 kb would completely discriminate *successes* from *fails*. The resulting leaves would therefore be “pure”: in one leave (LSS < 1,000 kb), all observations are *fails*, in the other one (LSS > 1,000 kb) all observations are *successes*. Such a node split uses only one feature to classify the observations according to their labels and the cut-point value that allowed this split is 1,000 kb. Node splits are determined automatically during tree growing, by going through all features and cut points and looking for the combination that minimizes the label impurity of the leaves defined by this combination. Label impurity reduction is quantified through a score measure, with the most common ones based on Gini index or information entropy (we use the former in our experiments). A complete decision tree is obtained by repeatedly applying these splitting operations on the whole learning sample until the resulting leaves are either pure (all examples they contain have the same label) or contain too few examples from the learning sample (this threshold is optimized by a parameter – see here below).

**Table 1:** Schematic example of a learning sample.

Features			Label
LSS (in kb)	GENGc	…	
100	0.51		Fail
1,500	−0.02		Success
350	0.49		Fail
400	0.36		Fail
15,000	0.52		Success
5,400	0.55		Success
240	0.04		Fail
850	0.38		Fail
350	0.44		Fail
400	0.45		Fail
15,000	0.44		Success
1,500	0.56		Success
350	0.32		Fail

*A target haplotype is compared to a panel of reference haplotypes at any LD map position. Two features (LSS, length of a shared segment; GENGc, genomic relationship between target and reference gamete on the current chromosome) are observed. Each observation can be a success (being the best matching reference haplotype at that position) or a fail, computed using HD map information.*

A single decision tree usually does not perform well in terms of predictive performance. Better results are obtained by aggregating the predictions, through a majority vote, of an ensemble of decision trees (called forests). Several ways to obtain the different decision trees that compose forests do exist. In Breiman’s (2001) original RF algorithm each tree is grown from a bootstrap sample drawn from the original learning sample and node splitting is modified so that the best split (feature and cut point) is searched within a random sample of *k* features, redrawn at each node. In contrast, in the extra-tree’s method, each tree is grown from the original learning sample without bootstrapping. When splitting a node, the best split is searched for among a subset of *k* randomly selected features like in standard RF, with the difference that the cut-point for each feature is selected randomly instead of being optimized to reduce label impurity as in standard RF. Extra-trees have been shown to be competitive with classical RF in terms of predictive performances while being more computationally efficient because of the extra-randomization (Geurts et al., 2006). For our specific case, they have also proven to yield more accurate results than classical RF (see Supplementary Material S1).

In this study, we used the extra-tree classifier implemented as part of the Python SciKit-Learn package (Pedregosa et al., 2011). Among the seventeen parameters of this implementation of the classifier, two were set to a value different than the default one (*n_estimators*, the number of trees, was set to 200 and *min_samples_split*, the minimum number of examples required to split a node, was set to 1) and two were set to vary as they were influencing results more than other parameters during exploratory runs (unpublished results). The first one (*max_features*, the number *k* of features randomly selected at each node) was set to vary over the range of values [1, 2, 3, 4, 5] and the second one (*min_samples_leaf*, the minimum number of examples required at a leaf node) was set to vary over the range of values [50, 150, 250, 500, 1000, 1500, 2000, 2500].

After the learning stage, extra-trees return the importance of each feature, which is a measure of the total reduction of impurity brought by that feature within the forest. The higher the importance of a given feature in the forest, the more relevant this feature is in predicting the label. Therefore, importance values can be used afterward to rank the features from the most to the least relevant and to gain some understanding of the problem.

#### Optimization of Extra-Trees Parameters

To tune these parameters, we used a second internal cross-validation loop. More precisely, each of the 13 groups of the external cross-validation loop (outlined in Figure 2) is further divided into 12 subgroups. Each of these 12 subgroups are divided into target and reference panels in the same way as for the 13 groups of the outer loop (see Figure 2). For each of the 5-by-8 combinations of the *max_features* and *min_samples_leaf* parameters and for each of the 12 subgroups, all target haplotypes are modeled as a mosaic of reference haplotypes and imputed, and the comparison criteria *e*_A_ and *r*^2^ are computed. For each criterion, the combination of parameters yielding the best values over all twelve subgroups is retained as the optimal one, returning therefore the two best combinations (one per criterion) used for the parent cross-validation group. Such two-level cross-validation is necessary to avoid artificial inflation of results that might arise if we would have used the target panel from the cross-validation group in the optimization of parameters.

#### Building the Learning Samples

The learning sample of each of the 13 cross-validation groups is built by successively considering each one of the 84 reference individuals as a target. Therefore, two haplotypes considered as targets are matched to 166 haplotypes considered as references along the 2,321 positions of our LD map. The maximal number of labeled observations in the learning sample of the cross-validation group is thus close to 65 million (2,321 × 2 × 166 × 84). Handling such a large learning sample would be tricky computationally speaking. Furthermore, we expect much of it to be redundant, which is the reason why we have downsized the number of labeled observations to two fixed sizes of 100,000 and 1,000,000, randomly picked from the 65 million possibilities and, respectively, denoted as EXT-100k and EXT-1M hereafter.

#### Selection of Features

Features from which observations are made were selected during exploratory analyses (unpublished results) and are listed in Table 2. We have listed 30 of them and ordered them in three main types : (1) those gathering information about local similarity between haplotypes, (2) those estimating the relationships between individuals, gametes, and haplotypes, and (3) those outputted from other methods for locally matching haplotypes.

**Table 2:** List of all features investigated for use in the random forests framework, with their names and ranges of variation.

			Range	
Type	Name	Description	Min	Max
Features based on position along	POS	Position along the SNPs of the LD panel	1	*P*
the chromosome and local	NSS	Length (in #POS) of the shared segments	0	*P*
haplotype sharing (16 features)	R1-NSS	Ranking (standard^∗^) of the length (in #POS) of the shared segment	1	*R*
	R2-NSS	Ranking (dense^∗^) of the length (in #POS) of the shared segment	1	*R*
	DLN	Distance (in #POS) to the left edge of the shared segment + 1	0	*P* + 1
	DRN	Distance (in #POS) to the right edge of the shared segment + 1	0	*P* + 1
	DMN	Distance (in #POS) to the closest edge of the shared segment + 1	0	*P* + 1
	R1-LSS	Ranking (standard^∗^) of the physical length of the shared segment	1	*R*
	R2-LSS	Ranking (dense^∗^) of the physical length of the shared segment	1	*R*
	iDLN	Inverse of DLN, as 2-(DLN)^−1^ when DLN > 0; 0 otherwise	0	2
	iDRN	Inverse of DRN, as 2-(DRN)^−1^ when DRN > 0; 0 otherwise	0	2
	iDMN	Inverse of DMN, as 2-(DMN)^−1^ when DMN > 0; 0 otherwise	0	2
	LSS	Physical length of the shared segments (in kb)	0	*L*
	DLL	Physical distance to the left edge of the shared segment	0	*L*
	DRL	Physical distance to the right edge of the shared segment	0	*L*
	DML	Physical distance to the closest edge of the shared segment	0	*L*
Features based on estimation of	PEDI	Pedigree relationship between reference and target individuals	0	2
relationship (11 features)	PEDG	Pedigree relationship between reference and target gametes	0	1
	GENI	Genomic relationship (as in Yang et al., 2010) between reference and target individuals on all chromosomes	(n.b.)	
	GENG	Genomic relationship (as in Yang et al., 2010) between reference and target gametes on all chromosomes	(n.b.)	
	GENIc	Genomic relationship (as in Yang et al., 2010) between reference and target individuals on the current chromosome	(n.b.)	
	GENGc	Genomic relationship (as in Yang et al., 2010) between reference and target gametes on the current chromosome	(n.b.)	
	SIMI	Genomic similarity between reference and target individuals on all chromosomes	0	1
	SIMG	Genomic similarity between reference and target gametes on all chromosomes	0	1
	SIMIc	Genomic similarity between reference and target individuals on the current chromosome	0	1
	SIMGc	Genomic similarity between reference and target gametes on the current chromosome	0	1
	MNT	Minimum number of ties to join the reference and target gametes using the pedigree (equal to 100 when MNT > 99)	1	100
Features outputted from other	PBLM	Probability of IBD obtained by the HMM-HP-LD method	0	1
methods (3 features)	R2-PBLM	Ranking (dense^∗^) of reference haplotypes according to their PBLM	1	*R*
	MASW	Moving average of the number of switches between longest shared segments in the surrounding 5 Mb	0	(n.b.)

*^∗^Standard ranking is “1134” whereas dense ranking is “1123.” The dense ranking allows comparing a situation where many reference haplotypes are the local best match to a situation where only one is the local best match: in both cases the second top-ranked reference has a ranking equal to 2. nb: not bounded.*

Features of the first type contain information about local similarity between target and reference haplotypes, according to their position along the phased chromosome. The LD position itself is one of these features, as well as a group of features related to the size of the segment shared between reference and target haplotypes (expressed in number of SNPs, in kb, or ranked) and a group of features related to the position inside a shared segment, expressed as the distance to the edges of the segment. If target and reference haplotypes do not share a segment at a given position, only the LD position is non-zero; as no identity was observed, there are no shared segments and therefore their length and distance to their edges are set to zero.

**FIGURE 3:**
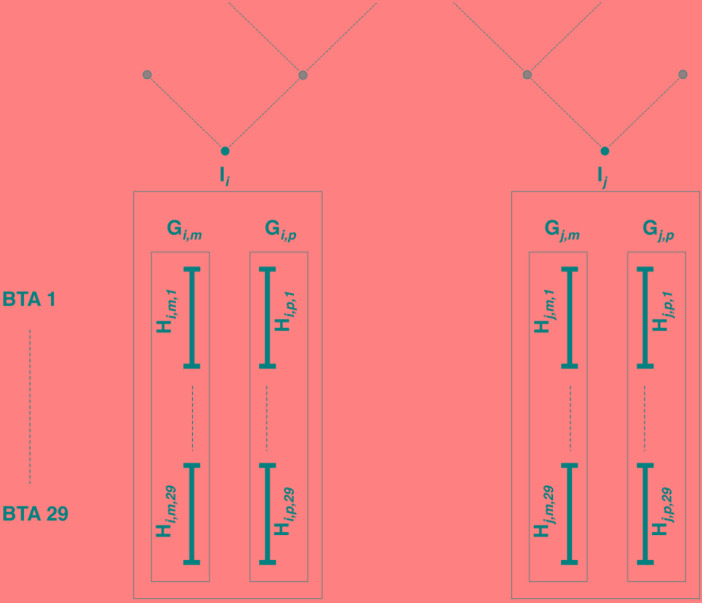
Estimated relationships between individuals, gametes, and haplotypes. Individuals *I*_i_ and *I*_j_ have been genotyped on the LD map; parental origins are known, and genotypes are accurately phased. All haplotypes inherited from mother (father) are denoted as maternal (paternal) gamete *G*_m_ (*G*_p_). For each of the 29 bovine autosomes, chromosomes are entirely phased in maternal (paternal) haplotypes *H*_m_ (*H*_p_).

Then come features related to (individual, gametic, haplotypic) relationships. Note that we understand the term “gamete” to mean the whole set of alleles inherited from each parent, as mentioned in previous studies involving gametic relationships (e.g., Schaeffer et al., 1989). Estimations are based on pedigree information and/or genomic information brought by the LD map. In the present study, haplotypes from individuals with ancestors in the sample are identified according to their parental origins (e.g., paternal vs. maternal haplotype). This allows the use of gametic relationships (e.g., based on the genealogy, the paternal haplotype is linked with both haplotypes from its father and eventually to haplotypes from paternal grand-parents, when these are present in the sample, but it is not linked to the haplotypes from its mother, assuming both parents are unrelated). Following notations in Figure 3, PEDI and PEDG are the additive relationships [estimated using pedigree information as defined in Wright (1922)], respectively, between individuals (e.g., *I*_i_ and *I*_j_) and gametes (e.g., *G*_i,p_ and *G*_j,p_, or *G*_i,p_ and *G*_j,m_). Genomic relationships (between individuals, gametes – on all autosomes – or haplotypes – only on current autosome and denoted with suffix “c”) are computed using the formula by Yang et al. (2010). That formula weights the relationship according to allelic frequencies. Conversely, the genomic similarities (between the same pairs of individuals, gametes, and haplotypes as for genomic relationships) do not take into account allelic frequencies (computed using Eq. 6 in Speed and Balding, 2014). Considering the pedigree as a directed graph, we have computed the feature MNT (for the *minimum number of ties*) as the shortest path from any gamete to another one.

Lastly come features outputted from other methods for locally matching haplotypes: (1) the probability that any reference haplotype would be the best local match haplotype for a given target haplotype (PBLM), as computed in our implementation of the HMM and ranked from highest to lowest (R1-PBLM), and (2) the average number of switches in the 5 Mb surrounding the current position (MASW), using a simple (unpublished) heuristic that reconstructs the target haplotype as a mosaic of segments from reference haplotypes under constraint of a minimal number of segments. Here, the rationale is that a high value of MASW could pinpoint a chromosomal region where no large reference haplotype could be assigned to the target haplotype. Through PBLM, the classifier is fed the data used by the HMM-HP-LD modality of our HMM (see the description here below, section “Modeling Target Haplotypes As a Mosaic of Reference Haplotypes”) without, however, specifying its selection rule (namely, the reference haplotype with the highest probability is chosen).

#### Tests With Reduced Number of Features

In order to better understand properties of the machine learning classifier, we have applied a similar evaluation protocol to four modalities corresponding to four relevant sets of features. Each of them was obtained from the learning samples used in the EXT-100k modality by hiding some features. EXT-100k-L contains all features from the first type (cf. Table 2), EXT-100k-LR contains all features from the first and second types, EXT-100k-H only contains the two features obtained from the HMM (PBLM and R1-PBLM) and the last one, EXT-100k-HR contains the two HMM features plus all features from the second type. In this case, the cross-validation plans, the comparison criteria and the learning samples are the same. The only difference lies in the range of tested values for optimization of the *max_features* parameter ([1, 2] instead of [1, 2, 3, 4, 5] to not exceed the number of features of the group with the lowest number of features).

#### Obtaining Evaluation Criteria

Once extra-trees have learnt discrimination rules using the learning sample, the rules are applied to unlabeled observations and, for any of them, the extra-tree classifier provides the probability that the observation belongs to the two score modalities: *P*_s_, the probability of *success*, complement *P*_f_, the probability of *fail*. For any target haplotype at any LD position, *P*_s_ are computed for each reference haplotype. The best match is the one that has obtained the highest (predicted) probability of success (in case of equality, the reference haplotype occurring at first in the vector of probability is chosen). Doing so for each LD position results in modeling the target haplotype as a mosaic of segments from the locally best matching reference haplotypes. The main criterion to assess the correctness of the mosaic target haplotype, the metric *e*_A_, is obtained by summing the difference of allelic content between a true target haplotype observed on the HD map and its modeling as a mosaic of HD segments from the reference haplotypes.

A first imputation of the target haplotypes (only observed on the LD map) may be achieved by considering the inferred mosaic of reference haplotypes (observed on both maps) on the HD map. However, haplotype imputation may yield better results if we consider more reference haplotypes rather than only the best matching one, e.g., if there are more than one best matching haplotype, or if some reference haplotypes have a *P*_s_ very close to the highest one. Therefore, we impute the allelic content ${\text{a}}_{\text{i}}^{\text{t}}$(a_i_ ∈ [0,1]) of a target haplotype *t* at SNP *i* by averaging over the allelic contents of all *Q* best-matching reference haplotypes among *R*(*Q* ≤ *R*) according to a weight *w_q_* as follows:

$$a_{i}^{\text{t}}=\sum_{q=1}^{\text{Q}}\left(w_{q}\cdot a_{i}^{\text{q}}\right)$$

The weight *w*_q_ is computed according to the probabilities of the best local match (*P*_s_) of the *Q* best-matching reference haplotypes at the LD position closest to HD position *i*:

$$w_{\text{q}}=\frac{P_{\text{s}(\text{q})}}{\sum_{\text{q}=1}^{\text{Q}}P_{\text{s}(\text{q})}}$$

The *Q* best-matching reference haplotypes are selected as those having a *P*_s_ greater or equal to a fraction c(c ∈ [0,1]) of the highest *P*_s_. For instance, setting *c* to 0 leads to a weighted average of all the *R* reference haplotypes. Nonetheless, such an option is not optimal: the best imputation results were obtained during exploratory runs with *c* close to 1.

For a given individual, the imputed HD dosages are obtained by summing the allelic contents of the two imputed haplotypes. Once genotype imputation is achieved for all animals, the imputation reliability (*r*^2^) can be computed at every HD map position. Note that the optimization of extra-tree parameters *max_features* and *min_samples_leaf* are independently achieved for each criterion chosen for comparison; optimized parameters, and thus optimized extra-trees, are different, whether the purpose was to optimize *e*_A_ or the imputation of *r*^2^. For imputation purposes, the value of *c* is optimized along with *max_features* and *min_samples_leaf* by setting it to vary in the range [0.75, 0.80, 0.85, 0.90, 0.95, 1.00].

### Hidden Markov Model for Local Haplotype Matching

#### Modeling Target Haplotypes as a Mosaic of Reference Haplotypes

IMPUTE2 (Howie et al., 2009) returns imputed genotypes without providing information on the best matching reference haplotypes. To obtain the mosaic structure, we have implemented an HMM equivalent to IMPUTE2 and similar to models underlying other HMM-based methods, e.g., MaCH (phasing and imputation, Li et al., 2006) or ChromoPainter (local ancestry inference, Lawson et al., 2012). Our model corresponds to settings where genotypes are pre-phased, thus it does not include a phasing step, nor does it integrate phasing uncertainties. Working straight from phased haplotypes rather than genotypes makes the method comparable to the random forests framework.

In this HMM, we model each target haplotype as an unobserved mosaic of the *R* reference haplotypes (hidden states). Emission probabilities *P*_e_ correspond to the probability to observe allele *k* (*k* = 0|1) at a position *p* when the underlying hidden state is a reference haplotype *r* and accounts for genotyping errors. Denoting the probability of error as *P*_error_, *P*_e_ is equal to 1 − *P*_error_ if alleles are identical and to *P*_error_ if alleles are not identical. Between positions *p* and *p* + 1, separated by a distance *d*_p,p + 1_ (in cM), the probability of transition P_t;p,p+1_ from hidden state *r* to hidden state *s*(*r, s* ∈ [1, *R*]) is estimated as:

$$P_{\text{t};\text{p},\text{p}+1}=\{\begin{array}{cc} \begin{array}{c} 1/R\cdot \left(1-\exp\left(-N_{\text{g}}d_{\text{p},\text{p}+1}\right)\right)\\ \exp\left(-N_{\text{g}}d_{\text{p},\text{p}+1}\right)+1/R\cdot \left(1-\exp\left(-N_{\text{g}}d_{\text{p},\text{p}+1}\right)\right) \end{array} & \begin{array}{l} \text{if }r\neq s\\ \text{if }r=s \end{array} \end{array}$$

In the formula above, *N*_g_ is a parameter corresponding to the expected number of generations from the target haplotype to the reference haplotype. Since the maximum number of reference haplotypes is low in our case (*R* = 168 at maximum, see Figure 2), we do not restrict the space of hidden states.

At each position, we compute the probability that the reference haplotype *r* contributes to the unobserved mosaic structure of target haplotype *t* according to the HMM. That probability is later referred to as the “best local match probability” (for consistency with definition used for the random forests framework) and is computed with the forward–backward algorithm (described in Rabiner, 1989). This algorithm efficiently computes the probabilities over all possible sequences of unobserved states and conditionally on all observations and on the parameters of the model.

Inferring a discrete mosaic sequence is achieved in two ways: (1) HMM-VI, selecting the most likely mosaic sequence using the Viterbi algorithm (also described in Rabiner, 1989), or (2) HMM-HP, selecting the hidden state (reference haplotype) with highest probability at each map position. The HMM is trained on the two genetic maps, LD and HD, leading therefore to four mosaic sequences (HMM-VI-LD, HMM-VI-HD, HMM-HP-LD, HMM-HP-HD).

The parameters *P*_error_ and *N*_g_ of the so-defined HMM have been chosen to mimic at best the behavior of IMPUTE2 with option *allow_large_regions* and default parameters except for *k_hap* (set to 168) and *Ne* (set to 200). The selected values are *P*_error_ = 0.0005 and *N*_g_ = 4.7619. The model was then applied to all 14 target haplotypes of each of the 13 cross-validation groups (see Figure 2).

#### Imputation of Target Haplotypes and Genotypes Using the HMM

For any map position, haplotype imputation of a given target haplotype is obtained by averaging the allelic content of all reference haplotypes according to their respective best local match probability (computed using forward–backward algorithm). When the HMM is trained on the LD map, HD positions that are unobserved on that map are imputed using probabilities computed at the closest LD positions. Imputed haplotypes are eventually paired per individual to yield imputed dosages. With the aforementioned values for parameters *P*_error_ and *N*_g_ and trained on the HD map, our implementation of the model behaves similarly enough to IMPUTE2 (using option *allow_large_regions* and the fore-mentioned values for parameters *k_hap* and *Ne*) to consider them as identical imputation methods (see correlations between imputation methods in Supplementary Material S2). Hereafter, genotype imputation results using the HD map are obtained by running IMPUTE2 (with fore-mentioned parameters) and results using the LD map are obtained by running our implementation of the HMM (denoted HMM-LD and written in Fortran 90).

## Results

### Importance of Features

**FIGURE 4:**
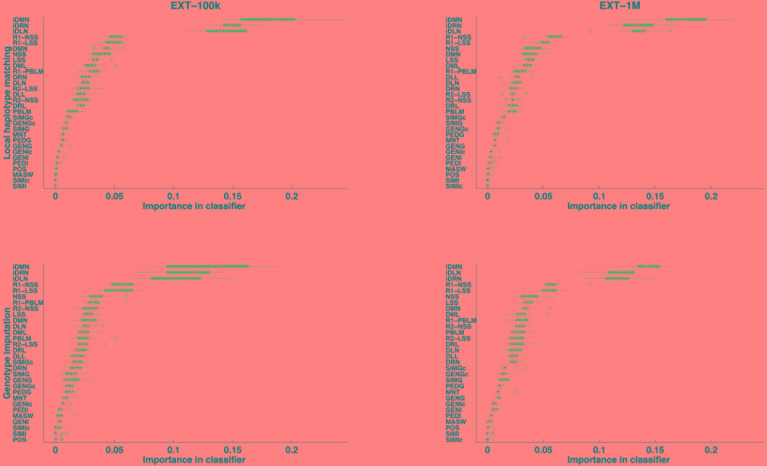
Features ranked by their importance in extra-trees (averaged over 13 cross-validations), for the purpose of locally matching haplotypes (top) or genotype imputation (bottom) and for two sizes of learning sample (100,000 and 1,000,000 labeled observations).

After supervised learning on the learning samples of the 13 cross-validation groups (see Figure 2), the importance of each of the 30 features was computed and averaged over the 13 cross-validation groups. The features are ranked by importance in Figure 4, for each case of size of learning sample and each purpose (inference of a target haplotype as a mosaic of reference haplotypes and genotype imputation from LD to HD map). The ranking is quite conserved between the four cases: from 96.9 to 99.7% of Spearman’s correlation, less correlated between purposes than between sizes of LS. The three top-ranked features are always iDMN, iDRN, and iDLN, three features expressing the distance to the edge of a shared segment (respectively the minimal, right and left distances) on an inverse scale. These three features mostly form a top group, well delimited from other features. It may be worth noting that those three features are always preferred to their corresponding ones on the regular scale (DMN, DRN, and DLN). Those are ranked in a second group of importance, alongside features related to the size of shared segments (NSS, LSS and their rankings). Features related to estimation of relationships (between gametes or individuals) are always low in rankings: SIMGc earns the highest ranking (17th) for a feature of this kind, ∼22 times less important than iDMN in that ranking. About features related to other assignation methods, the ranking of the best local match probability (R1-PBLM) is always more important than the probability itself (PBLM). The estimated number of switches in the neighboring 5 Mb (MASW) is consistently the least important feature, in the bottom group along with similarity between individuals.

**FIGURE 5:**
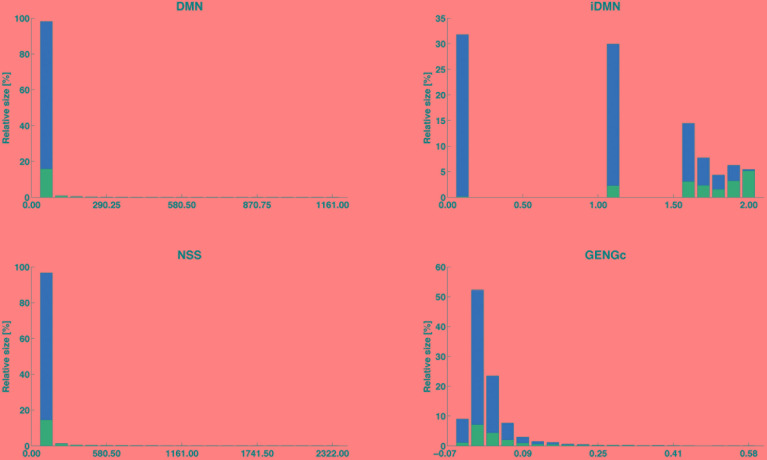
Distribution of the *success* labels along the ranges of four selected features. The range of each feature is divided into 20 equally spaced bins; the relative size of each bin (in %) is given by its height and its proportion of observations labeled with success is blue faced. The four features are DMN [distance (in #POS) to the closest edge of the shared segment +1], iDMN [inverse of DMN, as 2-(DMN)^−1^ when DMN > 0; 0 otherwise], NSS [length (in #POS) of the shared segments], and GENGc (genomic relationship between reference and target gametes, on the current chromosome).

The distribution of four selected features (iDMN, DMN, NSS, and GENGc) are given in Figure 5 (the detailed information is given in Supplementary Material S3). In that figure, the range of each of these features is divided in 20 equally spaced bins. The relative size of each bin is then computed as the proportion of observations falling into this bin. Among those observations, some are labeled with *success* (in blue), others with *fail* (in red). The purity of the bin is measured by the proportion of objects in this bin and labeled with *success*. This figure therefore shows how each of these four features is linked to the label. For each of them, the lower the value of the feature, the lower the purity and the larger the bins. However, feature iDMN reaches a better compromise between purity and size than feature GENGc does, for instance: less than 1% of the observations fall in the last bin of GENGc, in which 99.9% of the observations are successes, whereas 5.5% of the observations fall in the last bin of iDMN, in which purity is reasonably high (94.5% of the observations are successes). This may explain why iDMN is a good feature for classification.

### Differences Between True Haplotypes and Haplotypes Predicted Using Extra-Trees or the HMM

The 182 target haplotypes were modeled (per group of 14, see the cross-validation plan in Figure 2) as mosaics of HD segments from the best matching reference haplotypes. The metric *e*_A_ was then measured by comparing the modeled haplotypes to their known phase, for the four modalities of the HMM and the two modalities of the random forests framework. Results are averaged over the 182 haplotypes in Table 3. On these results, we see that the extra-trees classifier performs better than the other methods, whether the learning sample contains 1E5 or 1E6 objects. When a target haplotype is inferred as a mosaic of HD segments from the reference haplotypes that are locally classified as the best match, 98.75–98.77% of the HD positions have allelic content identical to the known target haplotype on the HD map. The HMM-HP-xx returns a lower median value than the extra-trees classifier; that median value difference is, however, much lower than the average difference.

**Table 3:** Inference of target haplotype as a mosaic of reference haplotypes.

	*e*_A_ [%]				Number of switches in inferred mosaic			
	Min	Avg	Med	Max	Min	Avg	Med	Max
HMM-VI-LD	**0.004**	1.441	0.430	11.936	**0**	15.7	**9.0**	73
HMM-HP-LD	0.005	1.304	0.413	7.401	**0**	19.5	**9.0**	91
HMM-VI-HD	0.005	1.413	0.409	8.327	**0**	**14.9**	**9.0**	**67**
HMM-HP-HD	0.005	1.310	**0.394**	7.403	**0**	27.6	**9.0**	671
EXT-100k	0.005	**1.226**	0.410	**6.941**	4	70.5	47.0	285
EXT-1M	0.006	1.231	0.414	7.026	4	95.8	71.0	367

*Distribution of the difference between predicted and true haplotypes (e_A_) and of the number of switches in the mosaic, on 182 haplotypes and 328,045 HD SNPs. Best results are boldfaced.*

Among the four HMM mosaic sequences, the method for selection of the local reference haplotype has more impact than that of the map on which the HMM was trained. Building the mosaic by selecting the hidden states (reference haplotypes) with the highest best local match probability (HMM-HP-xx) performs better on both maps than by selecting the best mosaic sequence with the Viterbi algorithm (HMM-VI-xx).

Methods are ranked almost reversely when looking at the number of switches in the mosaic in Table 3: the best mosaic sequences on *e*_A_ tend to model the target haplotype with more segments. For instance, when using the HMM, the mosaic obtained by the Viterbi algorithm (HMM-VI-xx) is less prone to switches than the mosaic obtained by selecting the reference haplotype with highest best local match probability (HMM-HP-xx), whatever the map (VI does 19 and 46% less switches than HP, respectively, for LD and HD maps). Conversely, the HP mosaic sequences have a lower proportion of error than the VI mosaic sequences (e.g., the average *e*_A_ is equal to 1.41% for HMM-VI-HD and 1.31% for HMM-HP-HD).

### Comparisons of Imputation Reliability Between Extra-Trees and HMM

In Table 4, results of imputation from LD to HD maps are detailed for the four methods of imputation: HMM using LD and HD maps (respectively HMM-LD and IMPUTE2) and extra-trees with 100,000 and 1,000,000 observations in the learning samples (respectively EXT-100k and EXT-1M). The imputation *r*^2^ are categorized by minor allele frequency (MAF) and position along the BTA1 chromosome. These results show that the extra-trees classifier performs as good as HMM: extra-trees classifiers are better on average imputation *r*^2^ whilst IMPUTE2 has a greater number of variants that are better imputed (higher median). Although slightly better on rare variants (MAF < 0.05) and between first and last Mb of the chromosome, the machine learning model is distinctly better than the HMM on chromosome edges: SNPs located on the last Mb of BTA1 have an average imputation *r*^2^ 2.23% higher for the best extra-trees (EXT-100k) than for the best HMM (IMPUTE2).

**Table 4:** Genotype imputation of target haplotypes.

		Overall	NMA^1^ = 2	MAF < 0.05	MAF > = 0.05	First Mb	Last Mb	Between first and last Mb	Number of SNP imputed as monomorphic
			
	*N*	325,358	4,020	41,931	283,427	2,587	2,370	320,401	
HMM-LD	Avg	91.86	71.89	80.96	93.47	87.89	87.74	91.92	125
	Med	94.93	99.15	90.22	95.04	92.61	90.30	95.00	
IMPUTE2	Avg	91.93	71.85	81.00	93.55	87.91	87.76	92.00	157
	Med	94.97	99.14	90.20	95.10	92.21	90.39	95.03	
EXT-100k	Avg	92.01	72.31	**81.52**	93.56	88.74	**89.99**	92.05	455
	Med	94.89	99.43	90.65	95.00	92.51	93.34	94.94	
EXT-1M	Avg	**92.08**	**72.33**	81.50	**93.65**	**89.28**	89.60	**92.12**	444
	Med	94.94	99.43	91.16	95.08	92.48	92.89	95.00	

*Average and median imputation r^2^ (as percentages) of four different imputation methods, partitioned by allele frequency and by position on BTA1, after exclusion of LD SNPs as well as any SNP imputed as monomorphic by at least one of the four methods. For each partition, the best average result is boldfaced. ^1^NMA, number of occurrences of Minor allele.*

The statistics in Table 4 relate to the SNPs that do not pertain to the LD map and for which imputation reliability was always computable (for that reason, SNPs imputed as monomorphic by one of the four methods were excluded). The numbers of SNP excluded for being imputed as monomorphic are proportionally very low (0.14% of the total number of only HD SNPs) but the random forests framework has imputed SNPs as monomorphic ∼3 to ∼4 times more than the HMM.

**FIGURE 6:**
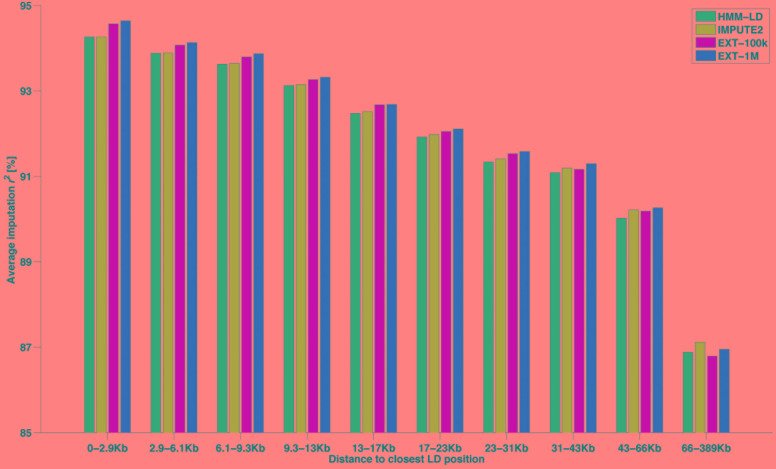
Average imputation *r*^2^ by four methods with regard to the distance between the imputed SNP (from the HD map) and the closest observed SNP (from the LD map), for different classes of distance containing the same number of imputed SNP.

Another way of categorizing SNPs to highlight imputation differences between methods is given in Figure 6. That figure shows the average imputation *r*^2^ in regard to the distance between the imputed HD SNP and the closest observed LD SNP. Ten classes of distance (from 0–2.9 to 66–389 kb) were designed so that they all include the same number (∼33k) of HD SNPs. For the HMM-based imputations, the figure shows that both maps return an equal average reliability up to ∼13 kb and then the HD map (IMPUTE2) overtakes the LD map (HMM-LD). Besides, whatever the size of the learning sample (EXT-100k or EXT-1M), the random forests framework always imputes better than the HMM which uses the same map (HMM-LD). As a result of these two trends, the random forests framework always yields better results than the HMM, except for the most distant class (>66 kb), where IMPUTE2 overtakes it. However, in that last distance class, the average imputation *r*^2^ drops for all methods.

#### Machine Learning With Reduced Number of Features

The results (Table 5) obtained when considering only the features of the first type (i.e., those based on the position along the chromosome) are quite close to the results obtained with all features, much more for inferring the target haplotype as a mosaic of segments than for genotype imputation. Adding the eleven relationship features further enhances these results. Note that the differences between Tables 3, 4 on average imputation *r*^2^ for a given method are due to the exclusion of more SNPs in Table 5, for being imputed as monomorphic in at least one of the tests.

**Table 5:** Effect of considering only some features and not others, on average difference *e*_A_ between predicted and true target haplotypes and on average imputation *r*^2^.

	*e*_A_	*r*^2^	Number of SNP imputed as monomorphic
		
*N*	182	324,738	
HMM-HP-LD| HMM-LD	1.304	92.00	**125**
HMM-HP-HD| IMPUTE2	1.310	92.07	157
EXT-100k	**1.236**	**92.15**	455
EXT-100k-L	1.240	91.73	577
EXT-100k-LR	1.238	91.83	692
EXT-100k-H	1.304	91.47	613
EXT-100k-HR	1.345	91.03	914

*Both comparison criteria are given as percentages and best results are boldfaced.*

Though lower, the results achieved by an automatic classifier only fed with two features – the features returned by the HMM (the probability of best local match and its ranking) – are still close to the “full” automatic classifier and actually slightly better than HMM-HP-HD for the purpose of inferring the target haplotype as a mosaic of segments. For that purpose, using the two HMM features with machine learning returns the same results as the HMM using the LD map (HMM-HP-LD). Surprisingly however, adding the relationship features yields worse results. The fact that the *max_features* parameter was set to vary between few and low values (1 or 2) could explain this unexpected result. For the purpose of imputation, considering only some features never reach average imputation reliabilities higher than those of the HMM.

## Discussion

### Genotype Imputation Illustrates the Effectiveness of the Random Forests Framework

When imputing WGS genotypes from 50k dense genotypes, the implemented random forests framework reaches average reliabilities similar to those achieved by IMPUTE2. We consider therefore these reliabilities as fair evidence of the ability of our framework to efficiently learn how to locally match haplotypes from examples (the labeled observations) for two main reasons. First, such a measure is independent of phasing, thus it does not embed potential phasing errors (even though those remain scarce). Second, using the imputation criterion makes it comparable to a state-of-the-art method, here IMPUTE2. Imputation results of the two types of methods are very similar, although we observed two main differences between HMM and the random forests framework. The first is that the random forests framework performs better on both edges of chromosomes: a difference of ∼2% of average imputation *r^2^* is observed. The second difference is that IMPUTE2 imputes genotypes at distant positions from known genotypes with higher accuracy; this is due to its use of the HD map, as shown by comparison with HMM-LD in Figure 6.

### Conceptual Differences Between the HMM and the Random Forests Framework

The differences in imputation results could be explained by the views behind the two types of methods, which also are quite distinct. The very basic conceptual difference between them lies in their modeling objectives: the HMM seeks to find the sequence of reference haplotypes that most likely reproduces an observed target haplotype (hence, essentially minimizing the number of segments) while our proposed framework searches for the best match locally (independently of the whole sequence). In some particular designs, the reference haplotypes correspond to the true ancestors of the target haplotype (e.g., Mott et al., 2000; Druet and Farnir, 2011; Zheng et al., 2015); then the HMM models the biological process of chromosomes transmission over a few generations. In contrast, the sequence returned by the random forests framework has no pretention to model that biological process but aims at imputing the target haplotype as well as possible, chunk after chunk. When the reference haplotypes are not the true ancestors of the target haplotype (e.g., when the target haplotype is not a true mosaic of reference haplotypes), the HMM framework no longer aims at finding the reference haplotype that is the most likely to be identical-by-descent (IBD) with the target haplotype at a given position but essentially minimizes the number of segments in the mosaic. Conversely, the random forests framework searches for the best match haplotype similarly to methods estimating IBD probability, considering the number of identical-by-state SNPs on both sides of the position (e.g., Meuwissen and Goddard, 2001). The natural consequence of these two different modeling purposes is a much higher level of “mosaicism” for the random forests framework (given in Table 3).

Beyond that first conceptual difference, another two are of interest. First, our framework does not allow for small differences between shared segments: a mismatch between target and reference haplotypes terminates a shared segment. For some methods (e.g., Beagle – Browning and Browning, 2009), more efficient imputation results have been observed without allowing differences. Not allowing differences also partially explains why the extra-trees makes more switches than the HMM. Note that the same constraint could be imposed in the HMM framework by setting *P_error_* to 0. Second, the two types of methods use different map information: the random forests framework only obtains information from the LD map whereas the HMM may additionally obtain information from the HD map. That difference matters since the HMM achieves better imputation with the HD map than with the LD map (particularly for HD SNPs distant from a LD position, see Figure 6). When it uses the entire map, the HMM better accounts for distances between SNP positions and for the structure of linkage disequilibrium between SNPs. It subsequently produces a better estimation of the haplotype blocks: a block is defined by SNPs in perfect linkage disequilibrium, not by those closest to a LD position. Integrating the information from the HD map into the random forests framework would therefore be profitable.

### Main Lessons of the Extra-Trees Classifier

Beyond its use, the random forests framework also reveals some useful lessons for the development of methods for local haplotype matching. The most informative lesson comes from the importance ranking of the features: top-ranked features are those expressing the distance to an edge of a shared segment (e.g., DMN, minimal distance to the left or right edge of the shared segment, or iDMN, its expression on an inverse scale). When such a feature is not equal to zero, it contains a double information: (1) that both haplotypes are, at this position, in a shared segment and (2) the value of the distance to the edges of the segment. A high value of DMN (or a value of iDMN close to 2) reveals that both haplotypes share a long identity segment (at least twice the length of the value of DMN) and that the current position is quite distant from the closest edge of this identity segment. The distance to the edge of a shared segment is thus more important than the length of this shared segment. As discussed above, the distance to the closest edge might better reflect relative local IBD probabilities than the length of the shared segment. Accordingly, minimizing the number of segments in the mosaic as done in the HMM does not guarantee the identification of the reference haplotype with the highest local IBD probability.

Before going further, note that the precedence of iDMN over DMN (and similarly for iDRN, iDLN) can be explained by the nature of extra-trees itself: for any node split when growing a decision tree, the extra-trees algorithm randomly picks up the value of the cut-point for a feature uniformly between the min and max value of this feature in the node to split. However, the sizes of classes of iDMN are more uniformly distributed over its (bounded) range than the sizes of classes of DMN (see Figure 5: for DMN, >98% of the observations fall into the first bin of range). Therefore, when picking at random a cut-point for node splitting, there is a higher chance of having an informative discrimination with iDMN compared with DMN. With classical random forests (where cut-points are optimized over the full range of values), iDMN and DMN have similar importance (see Supplementary Material S4).

Features rankings (Figure 4) also show that features of the first group (i.e., 16 features related to the position along the chromosome) unambiguously take the precedence over the ones of the second group (relationships). Such hierarchy was then confirmed by the tests with a reduced number of features (Table 5). This result was expected in the sense that the relationship features express identity between haplotypes at maximum at the chromosome level (feature GENGc, which actually is the most important of these features) whereas features from the first group express identity between haplotypes at a segment level (e.g., a high value of feature LSS reveals an identity spanning on several Mb). A second lesson is thus that relationship features have a small but not null impact: removing them from the random forests framework leads to average imputation reliabilities lower than those of the HMM (Table 5). Our explanation is that these relationships are still useful to discriminate between reference haplotypes bearing a shared segment of the same length, although for most of the cases the length of the shared segment already captures the familial information (long segments indicating close relationships). Consequently, using relationship to pre-select the subset of reference haplotypes, as done by SHAPEIT2 (Delaneau et al., 2011) or by LDMIP (Meuwissen and Goddard, 2010), is probably already a good way to use this information. Similarly, we observed that adding the relationship information to the HMM information (in the random forests framework) did not improve our accuracy.

The rankings of features (Figure 4) bring other minor lessons about features expressing the same aspect, but in a different way. First, feature NSS is always preferred to feature LSS, whereas both express the length of a shared segment between target and reference haplotypes (respectively in number of LD map positions and in kb). Second, the dense rankings are of little help: standard rankings (“R1-”) always take precedence over them (“R2-”). The rationale behind the use of the dense rankings was to make comparable cases where many reference haplotypes were the best match to cases where only one reference haplotype was the best match. In both situations, with dense ranking (“1123”), the second-best reference haplotype is ranked second whereas, with standard ranking (“1134”), the second-best reference haplotype is ranked *n* + 1, where *n* is the number of best matching haplotypes.

### Perspectives and Improvements for Routine Use of the Random Forests Framework

As implemented in our study, the random forests framework is not computationally competitive compared to the existing HMM approaches. Hence, prior to a routine application, two entangled aspects have to be considered: how does one achieve routine predictions with higher accuracy, and with lower computational demand than the random forests framework as implemented so far? Both aspects can be circumscribed to the constitution of the learning samples, summarizing the previous question to reducing the dimensions of these learning samples (number of labeled observations per number of features) along with improving accuracy.

On the aspect of the number of features, the tests conducted in this study have shown that discarding features could lead to very limited losses of precision but should not be done in a group-wise manner. Now that the hierarchy of features have been established inside each group, some features could be trimmed off to avoid redundancy, i.e., giving preference to iDMN over DMN, to NSS over LSS, or to R1- over R2. For instance, an optimized set of features may also be obtained through recursive feature elimination (Guyon et al., 2002). Besides removing less important features, new ones could also be investigated. Note that preliminary investigations are, however, always necessary for new features; for instance, we had considered the gametic linkage (as estimated in Wang et al., 1995) but too few relationships were non-zero so that it was helpless to identify best local matches between haplotypes. The IBD probabilities, as estimated by Beagle (Browning and Browning, 2009) or LDMIP (Meuwissen and Goddard, 2010), could also be considered although the usefulness of such features might be hampered by the time requested for computing them. Other features to consider are the allele (as in Maples et al., 2013), the MAF and the position of HD SNPs. These features would extend the learning sample to all HD positions, which would undoubtedly be profitable for accuracy. Conversely, this would directly impact the computational aspect. For that reason, an intermediate solution would be to consider blocks of linkage disequilibrium of HD SNPs (and their allele, MAF and position) instead of operating on these HD SNPs. All lengths and distances could also be expressed on a different scale to account for the average number of generations between target and reference haplotypes as in the HMM framework (e.g., using genetic distances and the number of generations to estimate recombination probabilities).

The number of labeled observations is the second aspect to consider and should be optimized alongside the number of features. Our results show a limited improvement when using a learning sample 10-times larger (EXT-1M vs. EXT-100k). The number of labeled observations could therefore be reduced. In addition, their selection could be achieved in a wiser manner, e.g., selecting them in order to contain the most different examples rather than randomly. The problem of the selection of the best training examples is known as active learning in machine learning literature (Settles, 2012).

## Conclusion

We herein outlined a new framework for automatically matching haplotypes along the chromosome and have illustrated that extremely randomized trees can effectively combine multiple sources of information to identify the best matching reference haplotypes. As an example, our implementation of the extremely randomized trees achieved slightly better imputation results than IMPUTE2. The random forests framework also allows identifying which features are the most important for a specific prediction. In the present case, distance to the edges of the shared segment appeared as the most important variable and adding genomic relationships only marginally improved results. To conclude, this approach might be further enhanced, for instance by including additional features, or could also be applied to other related applications such as identification of carriers of genetic defects or imputation of structural variants (by including features as distance with known carriers, genotyping intensity, etc.).

## Author Contributions

PF, PG, and TD conceived the study, interpreted the results, and wrote the manuscript. PF and TD developed the tools and software. PF carried out the experiments. All authors read and approved the final manuscript.

## Conflict of Interest Statement

The authors declare that the research was conducted in the absence of any commercial or financial relationships that could be construed as a potential conflict of interest.

## References

[B1] Baran Y. Pasaniuc B. Sankararaman S. Torgerson D. G. Gignoux C. Eng C. (2012). Fast and accurate inference of local ancestry in Latino populations. *Bioinformatics* 28 1359–1367. 10.1093/bioinformatics/bts144 22495753 PMC3348558

[B2] Breiman L. (2001). Random forests. *Mach. Learn.* 45 5–32.

[B3] Browning B. L. Browning S. R. (2009). A unified approach to genotype imputation and haplotype-phase inference for large data sets of trios and unrelated individuals. *Am. J. Hum. Genet.* 84 210–223. 10.1016/j.ajhg.2009.01.005 19200528 PMC2668004

[B4] Burdick J. T. Chen W.-M. Abecasis G. R. Cheung V. G. (2006). In silico method for inferring genotypes in pedigrees. *Nat. Genet.* 38 1002–1004. 10.1038/ng1863 16921375 PMC3005330

[B5] Charlier C. Li W. Harland C. Littlejohn M. Coppieters W. Creagh F. (2016). NGS-based reverse genetic screen for common embryonic lethal mutations compromising fertility in livestock. *Genome Res.* 26 1333–1341. 10.1101/gr.207076.116 27646536 PMC5052051

[B6] Daetwyler H. D. Wiggans G. R. Hayes B. J. Woolliams J. A. Goddard M. E. (2011). Imputation of missing genotypes from sparse to high density using long-range phasing. *Genetics* 189 317–327. 10.1534/genetics.111.128082 21705746 PMC3176129

[B7] Delaneau O. Marchini J. Zagury J.-F. (2011). A linear complexity phasing method for thousands of genomes. *Nat. Methods* 9 179–181. 10.1038/nmeth.1785 22138821

[B8] Druet T. Farnir F. P. (2011). Modeling of identity-by-descent processes along a chromosome between haplotypes and their genotyped ancestors. *Genetics* 188 409–419. 10.1534/genetics.111.127720 21441215 PMC3122309

[B9] Druet T. Georges M. (2010). A hidden markov model combining linkage and linkage disequilibrium information for haplotype reconstruction and quantitative trait locus fine mapping. *Genetics* 184 789–798. 10.1534/genetics.109.108431 20008575 PMC2845346

[B10] Faux P. Druet T. (2017). A strategy to improve phasing of whole-genome sequenced individuals through integration of familial information from dense genotype panels. *Genet. Sel. Evol.* 49:46. 10.1186/s12711-017-0321-6 28511677 PMC5434521

[B11] Geurts P. Ernst D. Wehenkel L. (2006). Extremely randomized trees. *Mach. Learn.* 63 3–42. 10.1007/s10994-006-6226-1

[B12] Guyon I. Weston J. Barnhill S. Vapnik V. (2002). Gene selection for cancer classification using support vector machines. *Mach. Learn.* 46 389–422.

[B13] Hastie T. Tibshirani R. Friedman J. H. (2017). *The Elements of Statistical Learning: Data Mining, Inference, and Prediction. 2nd edition, Corrected at 12th Printing.* New York, NY: Springer.

[B14] Howie B. N. Donnelly P. Marchini J. (2009). A flexible and accurate genotype imputation method for the next generation of genome-wide association studies. *PLoS Genet.* 5:e1000529. 10.1371/journal.pgen.1000529 19543373 PMC2689936

[B15] Kong A. Masson G. Frigge M. L. Gylfason A. Zusmanovich P. Thorleifsson G. (2008). Detection of sharing by descent, long-range phasing and haplotype imputation. *Nat. Genet.* 40 1068–1075. 10.1038/ng.216 19165921 PMC4540081

[B16] Lawson D. J. Hellenthal G. Myers S. Falush D. (2012). Inference of population structure using dense haplotype data. *PLoS Genet.* 8:e1002453. 10.1371/journal.pgen.1002453 22291602 PMC3266881

[B17] Li Y. Ding J. Abecasis G. R. (2006). Mach 1.0: rapid haplotype reconstruction and missing genotype inference. *Am. J. Hum. Genet.* 79:S2290.

[B18] Libbrecht M. W. Noble W. S. (2015). Machine learning applications in genetics and genomics. *Nat. Rev. Genet.* 16 321–332. 10.1038/nrg3920 25948244 PMC5204302

[B19] Maples B. K. Gravel S. Kenny E. E. Bustamante C. D. (2013). RFMix: a discriminative modeling approach for rapid and robust local-ancestry inference. *Am. J. Hum. Genet.* 93 278–288. 10.1016/j.ajhg.2013.06.020 23910464 PMC3738819

[B20] Marchini J. Howie B. Myers S. McVean G. Donnelly P. (2007). A new multipoint method for genome-wide association studies by imputation of genotypes. *Nat. Genet.* 39 906–913. 10.1038/ng2088 17572673

[B21] Meuwissen T. Goddard M. (2010). The use of family relationships and linkage disequilibrium to impute phase and missing genotypes in up to whole-genome sequence density genotypic data. *Genetics* 185 1441–1449. 10.1534/genetics.110.113936 20479147 PMC2927768

[B22] Meuwissen T. M. H. Goddard M. E. (2001). Prediction of identity by descent probabilities from marker-haplotypes. *Genet. Sel. Evol.* 33 605–634. 10.1051/gse:2001134 11742632 PMC2705394

[B23] Mott R. Talbot C. J. Turri M. G. Collins A. C. Flint J. (2000). A method for fine mapping quantitative trait loci in outbred animal stocks. *Proc. Natl. Acad. Sci. U.S.A.* 97 12649–12654. 10.1073/pnas.230304397 11050180 PMC18818

[B24] Pedregosa F. Varoquaux G. Gramfort A. Michel V. Thirion B. Grisel O. (2011). Scikit-learn: machine learning in python. *J. Mach. Learn. Res.* 12 2825–2830.

[B25] Price A. L. Tandon A. Patterson N. Barnes K. C. Rafaels N. Ruczinski I. (2009). Sensitive detection of chromosomal segments of distinct ancestry in admixed populations. *PLoS Genet.* 5:e1000519. 10.1371/journal.pgen.1000519 19543370 PMC2689842

[B26] Rabiner L. (1989). A tutorial on hidden Markov models and selected applications in speech recognition. *Proc. IEEE* 77 257–286. 10.1109/5.18626

[B27] Sargolzaei M. Chesnais J. P. Schenkel F. S. (2014). A new approach for efficient genotype imputation using information from relatives. *BMC Genomics* 15:478. 10.1186/1471-2164-15-478 24935670 PMC4076979

[B28] Schaeffer L. R. Kennedy B. W. Gibson J. P. (1989). The inverse of the gametic relationship matrix. *J. Dairy Sci.* 72 1266–1272. 10.3168/jds.s0022-0302(89)79231-6

[B29] Scheet P. Stephens M. (2006). A fast and flexible statistical model for large-scale population genotype data: applications to inferring missing genotypes and haplotypic phase. *Am. J. Hum. Genet.* 78 629–644. 10.1086/502802 16532393 PMC1424677

[B30] Settles B. (2012). Active learning. *Synth. Lect. Artif. Intell. Mach. Learn.* 6 1–114.

[B31] Speed D. Balding D. J. (2014). Relatedness in the post-genomic era: is it still useful? *Nat. Rev. Genet.* 16 33–44. 10.1038/nrg3821 25404112

[B32] Su Z. Cardin N. The Wellcome Trust Case Control Consortium Donnelly P. Marchini J. (2009). A bayesian method for detecting and characterizing allelic heterogeneity and boosting signals in genome-wide association studies. *Stat. Sci.* 24 430–450. 10.1214/09-STS311

[B33] Wang T. Fernando R. L. van der Beek S. Grossman M. von Arendonk J. (1995). Covariance between relatives for a marked quantitative trait locus. *Genet. Sel. Evol.* 27 251–274. 10.1186/1297-9686-27-3-251 11333832

[B34] Wright S. (1922). Coefficients of Inbreeding and relationship. *Am. Nat.* 56 330–338. 10.2307/2456273

[B35] Yang J. Benyamin B. McEvoy B. P. Gordon S. Henders A. K. Nyholt D. R. (2010). Common SNPs explain a large proportion of the heritability for human height. *Nat. Genet.* 42 565–569. 10.1038/ng.608 20562875 PMC3232052

[B36] Zheng C. Boer M. P. van Eeuwijk F. A. (2015). Reconstruction of genome ancestry blocks in multiparental populations. *Genetics* 200 1073–1087. 10.1534/genetics.115.177873 26048018 PMC4574238

