# Supplementary material for: A Random Forests Framework for Modeling Haplotypes as Mosaics of Reference Haplotypes
Source: Front Genet. 2019 Jun 27;10:562. doi: 10.3389/fgene.2019.00562 (PMC6610336; doi:10.3389/fgene.2019.00562)
Supplement: Supplementary file 1 [file Table_1.DOCX]

Supplementary Material

**A machine learning framework for modeling haplotypes as mosaics of reference haplotypes**

Pierre Faux^*^, Pierre Geurts, Tom Druet

*** Correspondence:** Pierre Faux: pierrefaux@gmail.com

# Supplementary Tables and Figures

## Supplementary Tables

**S1.** **Comparisons with classical random forests.** Average difference between predicted and true haplotypes (*e_A_*), average number of switches in the mosaic, average reliability of genotype imputation (*r*^2^, on all SNPs that were not imputed as monomorphic by none of the methods) and number of SNPs imputed as monomorphic for the HMM using both LD and HD maps and for the machine learning framework using either extra-trees (EXT-) or classical random forests (RAF-) on the same learning samples of two different sizes (100,000 or 1,000,000 labeled observations). Comparison criteria (*e_A_* and *r*^2^) are given as percentages and best results are boldfaced.

|  | ***e_A_*** | **Number of switches** | ***r*^2^** | **Number of SNPs imputed as monomorphic** |
| --- | --- | --- | --- | --- |
| ***N*** | 182 | 182 | 325,148 |  |
| **HMM-HP-LD \| HMM-LD** | 1.304 | **19.5** | 91.91 | **125** |
| **HMM-HP-HD \| IMPUTE2** | 1.310 | 27.6 | 91.98 | 157 |
| **EXT-100k** | 1.236 | 60.1 | 92.06 | 455 |
| **EXT-1M** | 1.223 | 57.9 | **92.13** | 444 |
| **RAF-100k** | 1.221 | 99.3 | 91.75 | 642 |
| **RAF-1M** | **1.217** | 94.6 | 91.73 | 678 |

**S2.** Correlations between imputation *r*^2^ (as percentages) of five different imputation modalities of two methods (HMM: hidden Markov model and EXT: extra-trees classifier), from LD to HD maps^1^.

|  | **HMM-LD** | **HMM-HD** | **IMPUTE2** | **EXT-100k** |
| --- | --- | --- | --- | --- |
| **HMM-HD** | 99.7941 |  |  |  |
| **IMPUTE2** | 99.7938 | 99.9999 |  |  |
| **EXT-100k** | 94.5642 | 94.6831 | 94.6831 |  |
| **EXT-1M** | 95.1389 | 95.2629 | 95.2640 | 98.3494 |

^1^After exclusion of any LD marker and any marker imputed as monomorphic in at least one of the five methods, correlations are computed over 325,358 markers.

**S3. Purity and weight of twenty classes of four different features (iDMN, NSS, DMN and GENGc) in thirteen learning samples of 1,000,000 objects each. The classes are uniformly distributed along the range of the values of the feature.**

| **CLASS#** | **iDMN** | | | | **DMN** | | | | **NSS** | | | | **GENGc** | | | |
| --- | --- | --- | --- | --- | --- | --- | --- | --- | --- | --- | --- | --- | --- | --- | --- | --- |
|  | *Inverse of DMN, as 2-(DMN)^-1^ when DMN>0; 0 otherwise* | | | | *Distance (in #POS) to the closest edge of the shared segment + 1* | | | | *Length (in #POS) of the shared segments* | | | | *Genomic relationship between reference and target gametes on the current chromosome* | | | |
|  | **from** | **to** | **class size** | **class purity** | **from** | **to** | **class size** | **class purity** | **from** | **to** | **class size** | **class purity** | **from** | **to** | **class size** | **class purity** |
| **1** | 0.0 | 0.1 | 31.8 | 0.5 | 0 | 58 | 98.1 | 16.2 | 0 | 116 | 96.7 | 15.0 | -0.07 | -0.04 | 9.0 | 11.9 |
| **2** | 0.1 | 0.2 | 0.0 | NaN | 58 | 116 | 0.8 | 98.5 | 116 | 232 | 1.3 | 95.5 | -0.04 | -0.01 | 52.3 | 13.6 |
| **3** | 0.2 | 0.3 | 0.0 | NaN | 116 | 174 | 0.4 | 98.9 | 232 | 348 | 0.5 | 97.5 | -0.01 | 0.02 | 23.5 | 18.6 |
| **4** | 0.3 | 0.4 | 0.0 | NaN | 174 | 232 | 0.2 | 98.6 | 348 | 464 | 0.3 | 98.3 | 0.02 | 0.06 | 7.7 | 25.6 |
| **5** | 0.4 | 0.5 | 0.0 | NaN | 232 | 290 | 0.2 | 98.7 | 464 | 581 | 0.3 | 98.2 | 0.06 | 0.09 | 2.9 | 32.2 |
| **6** | 0.5 | 0.6 | 0.0 | NaN | 290 | 348 | 0.1 | 98.5 | 581 | 697 | 0.2 | 98.2 | 0.09 | 0.12 | 1.5 | 38.0 |
| **7** | 0.6 | 0.7 | 0.0 | NaN | 348 | 406 | 0.1 | 98.7 | 697 | 813 | 0.2 | 98.0 | 0.12 | 0.15 | 1.1 | 45.0 |
| **8** | 0.7 | 0.8 | 0.0 | NaN | 406 | 464 | 0.1 | 98.8 | 813 | 929 | 0.2 | 98.3 | 0.15 | 0.19 | 0.6 | 48.9 |
| **9** | 0.8 | 0.9 | 0.0 | NaN | 464 | 522 | 0.0 | 98.6 | 929 | 1,045 | 0.1 | 99.0 | 0.19 | 0.22 | 0.5 | 54.4 |
| **10** | 0.9 | 1.0 | 0.0 | NaN | 522 | 580 | 0.0 | 98.5 | 1,045 | 1,161 | 0.1 | 98.2 | 0.22 | 0.25 | 0.2 | 58.5 |
| **11** | 1.0 | 1.1 | 29.9 | 7.6 | 581 | 639 | 0.0 | 98.5 | 1,161 | 1,277 | 0.1 | 97.4 | 0.25 | 0.28 | 0.2 | 67.4 |
| **12** | 1.1 | 1.2 | 0.0 | NaN | 639 | 697 | 0.0 | 99.1 | 1,277 | 1,393 | 0.1 | 98.9 | 0.28 | 0.32 | 0.2 | 70.3 |
| **13** | 1.2 | 1.3 | 0.0 | NaN | 697 | 755 | 0.0 | 99.1 | 1,393 | 1,509 | 0.0 | 98.6 | 0.32 | 0.35 | 0.1 | 79.9 |
| **14** | 1.3 | 1.4 | 0.0 | NaN | 755 | 813 | 0.0 | 98.7 | 1,509 | 1,625 | 0.0 | 97.7 | 0.35 | 0.38 | 0.1 | 83.8 |
| **15** | 1.4 | 1.5 | 0.0 | NaN | 813 | 871 | 0.0 | 99.6 | 1,625 | 1,742 | 0.0 | 99.3 | 0.38 | 0.41 | 0.1 | 88.1 |
| **16** | 1.5 | 1.6 | 14.4 | 21.3 | 871 | 929 | 0.0 | 98.6 | 1,742 | 1,858 | 0.0 | 99.1 | 0.41 | 0.45 | 0.0 | 83.2 |
| **17** | 1.6 | 1.7 | 7.7 | 29.9 | 929 | 987 | 0.0 | 99.2 | 1,858 | 1,974 | 0.0 | 94.0 | 0.45 | 0.48 | 0.0 | NaN |
| **18** | 1.7 | 1.8 | 4.4 | 35.5 | 987 | 1,045 | 0.0 | 99.0 | 1,974 | 2,090 | 0.0 | 99.6 | 0.48 | 0.51 | 0.0 | 91.1 |
| **19** | 1.8 | 1.9 | 6.3 | 51.0 | 1,045 | 1,103 | 0.0 | 97.4 | 2,090 | 2,206 | 0.0 | 97.6 | 0.51 | 0.54 | 0.0 | 97.1 |
| **20** | 1.9 | 2.0 | 5.5 | 94.5 | 1,103 | 1,161 | 0.0 | 97.3 | 2,206 | 2,321 | 0.0 | 97.6 | 0.54 | 0.58 | 0.0 | 99.9 |

The size of a class is the proportion of observations in the corresponding range.

The purity of a class is the proportion of observations in the corresponding range and labeled with *success*.

## Supplementary Figures

**Supplementary Figure S4.** Features ranked by their importance in classical random forests (averaged over 13 cross-validations), for the purpose of locally matching haplotypes (top) or genotype imputation (bottom) and for two sizes of learning sample (100,000 and 1,000,000 labeled observations.
